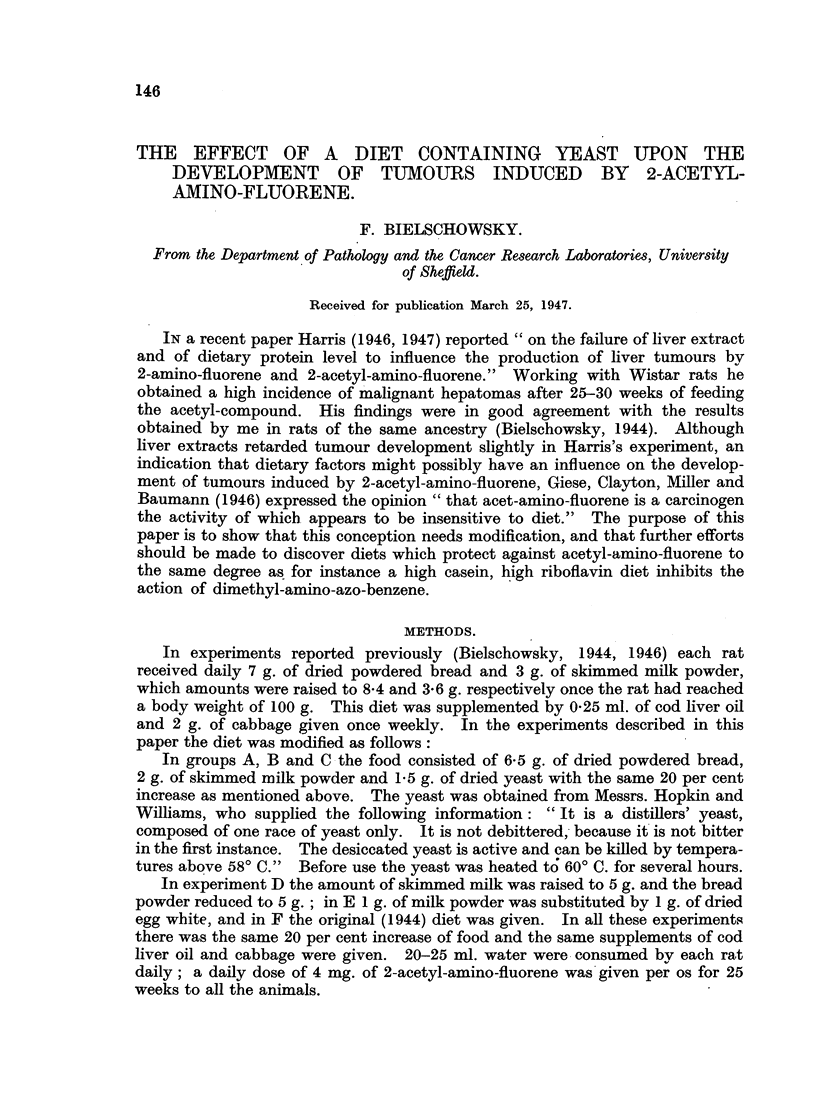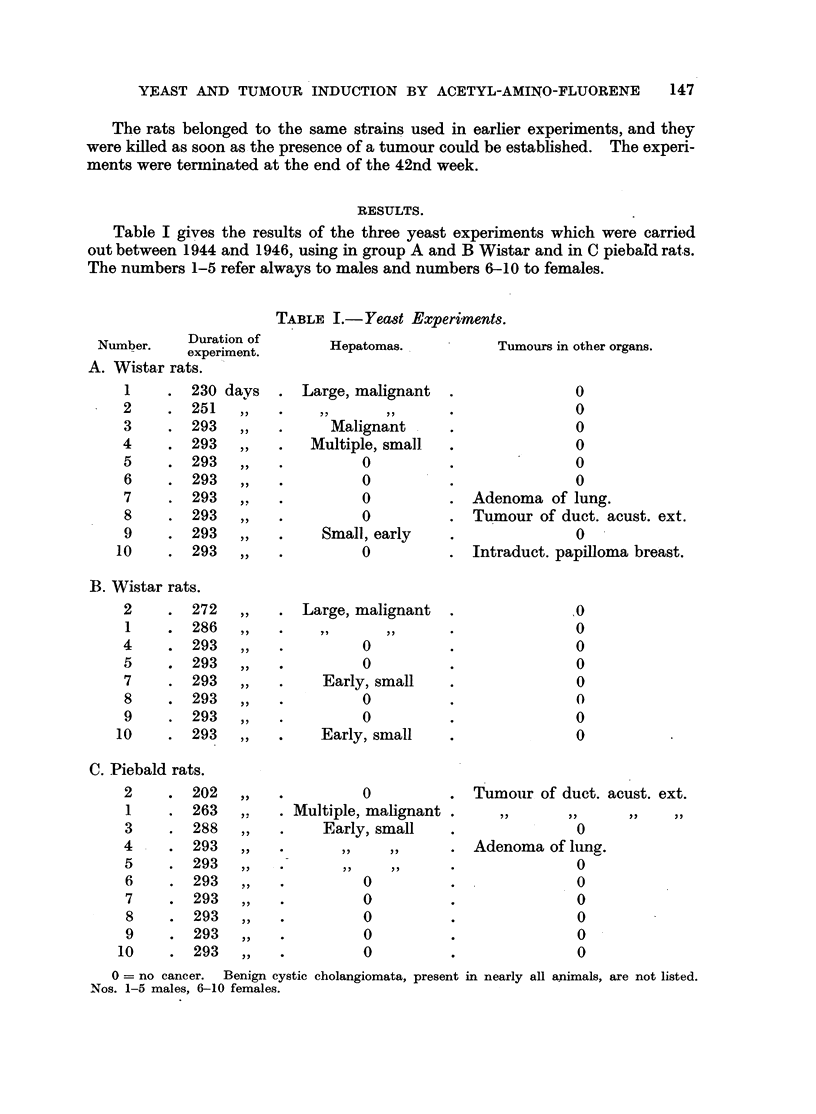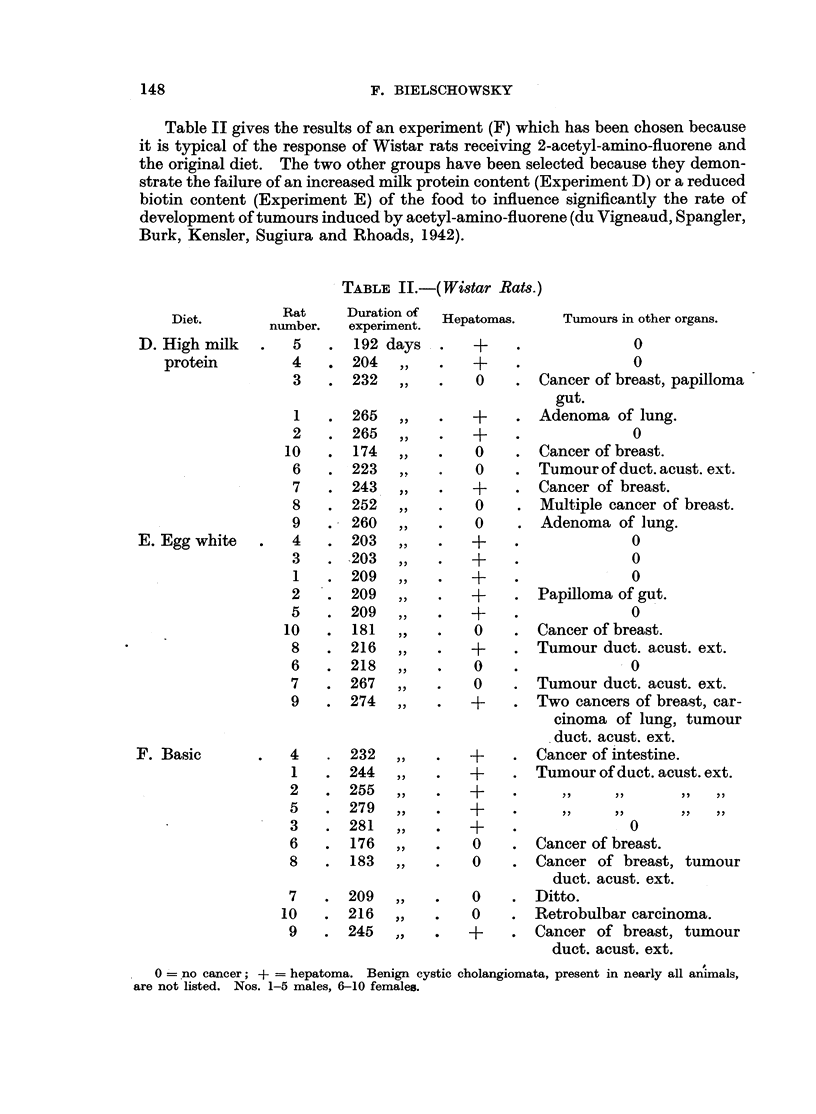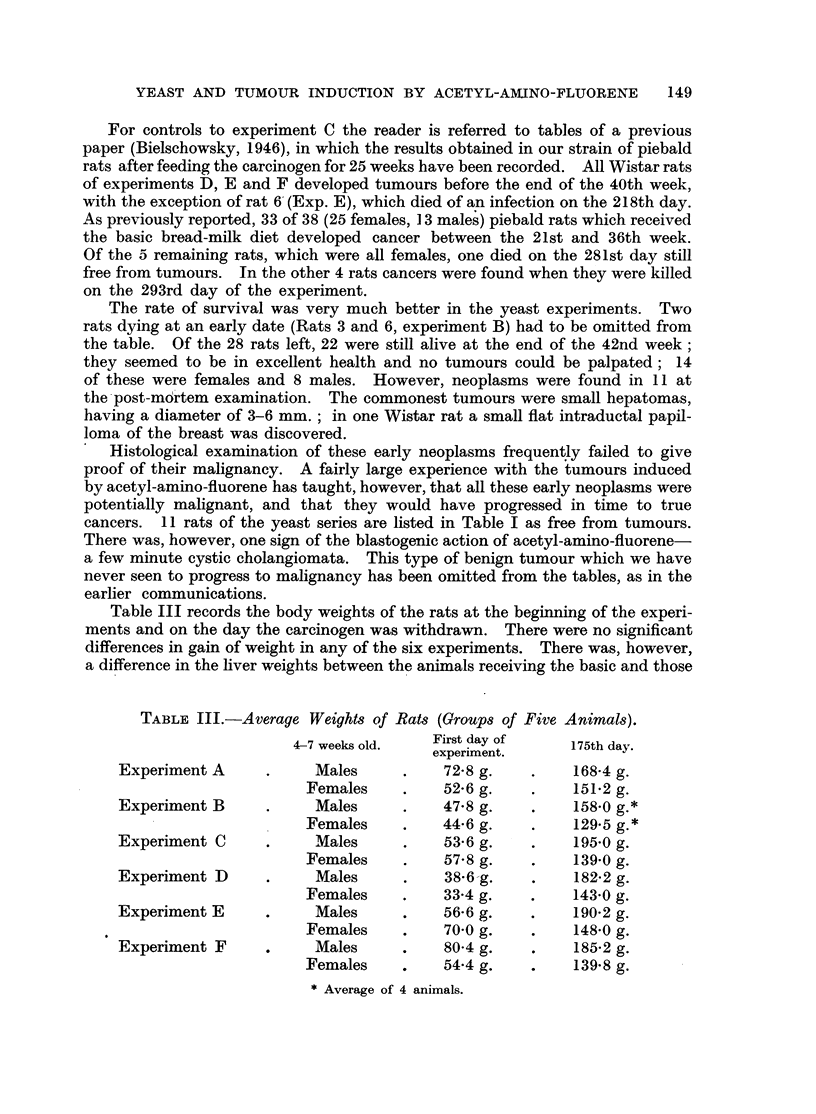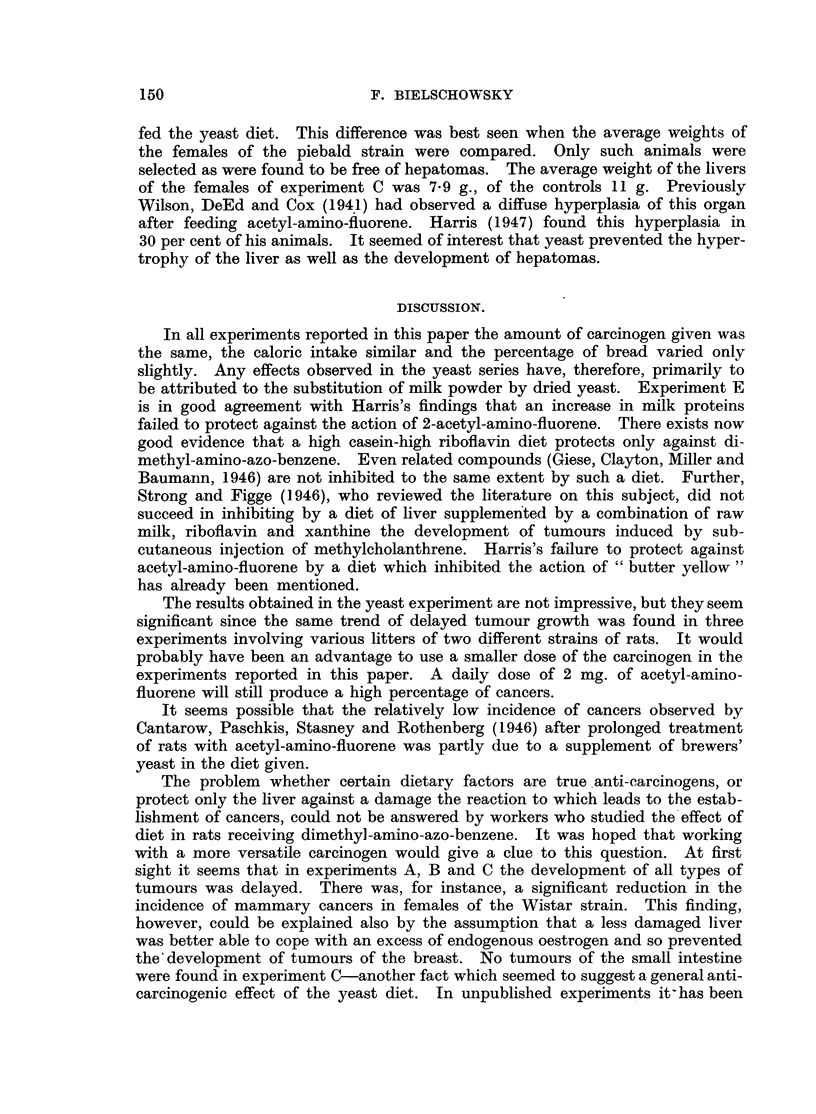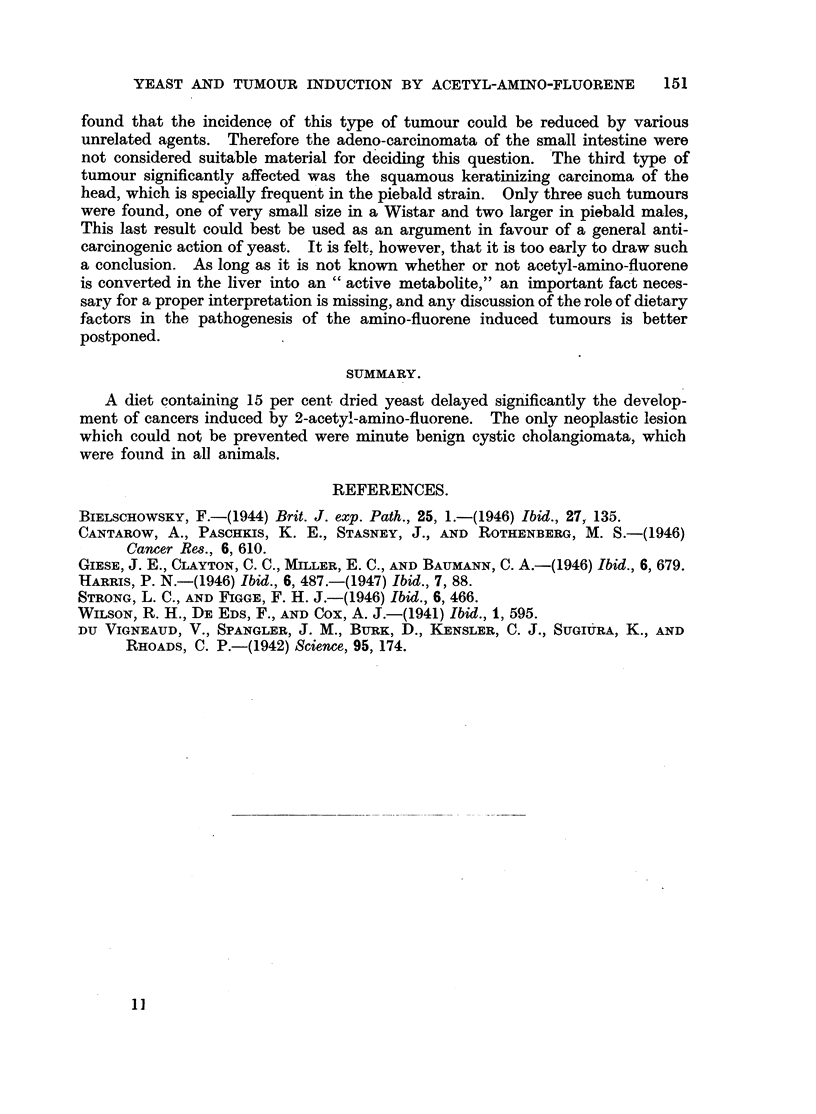# The Effect of a Diet Containing Yeast upon the Development of Tumours Induced by 2-Acetyl-amino-fluorene

**DOI:** 10.1038/bjc.1947.16

**Published:** 1947-06

**Authors:** F. Bielschowsky


### THE EFFECT OF A DIET CONTAINING YEAST UPON THE DEVELOPMENT OF TUMOURS INDUCED BY 2-ACETYL-AMINO-FLUORENE.

F. BIELSCHOWSKY.

*From the Department of Pathology and the Cancer Research Laboratories, University of Sheffield.*

Received for publication March 25, 1947.

IN a recent paper Harris (1946, 1947) reported " on the failure of liver extract and of dietary protein level to influence the production of liver tumours by 2-amino-fluorene and 2-acetyl-amino-fluorene." Working with Wistar rats he obtained a high incidence of malignant hepatomas after 25–30 weeks of feeding the acetyl-compound. His findings were in good agreement with the results obtained by me in rats of the same ancestry (Bielschowsky, 1944). Although liver extracts retarded tumour development slightly in Harris's experiment, an indication that dietary factors might possibly have an influence on the development of tumours induced by 2-acetyl-amino-fluorene, Giese, Clayton, Miller and Baumann (1946) expressed the opinion " that acet-amino-fluorene is a carcinogen the activity of which appears to be insensitive to diet." The purpose of this paper is to show that this conception needs modification, and that further efforts should be made to discover diets which protect against acetyl-amino-fluorene to the same degree as for instance a high casein, high riboflavin diet inhibits the action of dimethyl-amino-azo-benzene.

### METHODS.

In experiments reported previously (Bielschowsky, 1944, 1946) each rat received daily 7 g. of dried powdered bread and 3 g. of skimmed milk powder, which amounts were raised to 8·4 and 3·6 g. respectively once the rat had reached a body weight of 100 g. This diet was supplemented by 0·25 ml. of cod liver oil and 2 g. of cabbage given once weekly. In the experiments described in this paper the diet was modified as follows :

In groups A, B and C the food consisted of 6·5 g. of dried powdered bread, 2 g. of skimmed milk powder and 1·5 g. of dried yeast with the same 20 per cent increase as mentioned above. The yeast was obtained from Messrs. Hopkin and Williams, who supplied the following information : " It is a distillers' yeast, composed of one race of yeast only. It is not debittered, because it is not bitter in the first instance. The desiccated yeast is active and can be killed by temperatures above 58° C." Before use the yeast was heated to 60° C. for several hours.

In experiment D the amount of skimmed milk was raised to 5 g. and the bread powder reduced to 5 g. ; in E 1 g. of milk powder was substituted by 1 g. of dried egg white, and in F the original (1944) diet was given. In all these experiments there was the same 20 per cent increase of food and the same supplements of cod liver oil and cabbage were given. 20–25 ml. water were consumed by each rat daily ; a daily dose of 4 mg. of 2-acetyl-amino-fluorene was given per os for 25 weeks to all the animals.

The rats belonged to the same strains used in earlier experiments, and they were killed as soon as the presence of a tumour could be established. The experiments were terminated at the end of the 42nd week.

### RESULTS.

Table I gives the results of the three yeast experiments which were carried out between 1944 and 1946, using in group A and B Wistar and in C piebald rats. The numbers 1–5 refer always to males and numbers 6–10 to females.

TABLE I.—*Yeast Experiments.*

| Number. | Duration of experiment. | Hepatomas. | Tumours in other organs. |
|---|---|---|---|
| A. Wistar rats. | | | |
| 1 | 230 days | Large, malignant | 0 |
| 2 | 251 ,, | ,, ,, | 0 |
| 3 | 293 ,, | Malignant | 0 |
| 4 | 293 ,, | Multiple, small | 0 |
| 5 | 293 ,, | 0 | 0 |
| 6 | 293 ,, | 0 | 0 |
| 7 | 293 ,, | 0 | Adenoma of lung. |
| 8 | 293 ,, | 0 | Tumour of duct. acust. ext. |
| 9 | 293 ,, | Small, early | 0 |
| 10 | 293 ,, | 0 | Intraduct. papilloma breast. |
| B. Wistar rats. | | | |
| 2 | 272 ,, | Large, malignant | 0 |
| 1 | 286 ,, | ,, ,, | 0 |
| 4 | 293 ,, | 0 | 0 |
| 5 | 293 ,, | 0 | 0 |
| 7 | 293 ,, | Early, small | 0 |
| 8 | 293 ,, | 0 | 0 |
| 9 | 293 ,, | 0 | 0 |
| 10 | 293 ,, | Early, small | 0 |
| C. Piebald rats. | | | |
| 2 | 202 ,, | 0 | Tumour of duct. acust. ext. |
| 1 | 263 ,, | Multiple, malignant | ,, ,, ,, ,, |
| 3 | 288 ,, | Early, small | 0 |
| 4 | 293 ,, | ,, ,, | Adenoma of lung. |
| 5 | 293 ,, | ,, ,, | 0 |
| 6 | 293 ,, | 0 | 0 |
| 7 | 293 ,, | 0 | 0 |
| 8 | 293 ,, | 0 | 0 |
| 9 | 293 ,, | 0 | 0 |
| 10 | 293 ,, | 0 | 0 |

0 = no cancer. Benign cystic cholangiomata, present in nearly all animals, are not listed. Nos. 1–5 males, 6–10 females.

Table II gives the results of an experiment (F) which has been chosen because it is typical of the response of Wistar rats receiving 2-acetyl-amino-fluorene and the original diet. The two other groups have been selected because they demonstrate the failure of an increased milk protein content (Experiment D) or a reduced biotin content (Experiment E) of the food to influence significantly the rate of development of tumours induced by acetyl-amino-fluorene (du Vigneaud, Spangler, Burk, Kensler, Sugiura and Rhoads, 1942).

TABLE II.—(*Wistar Rats.*)

| Diet. | Rat number. | Duration of experiment. | Hepatomas. | Tumours in other organs. |
|---|---|---|---|---|
| D. High milk protein | 5 | 192 days | + | 0 |
| | 4 | 204 ,, | + | 0 |
| | 3 | 232 ,, | 0 | Cancer of breast, papilloma gut. |
| | 1 | 265 ,, | + | Adenoma of lung. |
| | 2 | 265 ,, | + | 0 |
| | 10 | 174 ,, | 0 | Cancer of breast. |
| | 6 | 223 ,, | 0 | Tumour of duct. acust. ext. |
| | 7 | 243 ,, | + | Cancer of breast. |
| | 8 | 252 ,, | 0 | Multiple cancer of breast. |
| | 9 | 260 ,, | 0 | Adenoma of lung. |
| E. Egg white | 4 | 203 ,, | + | 0 |
| | 3 | 203 ,, | + | 0 |
| | 1 | 209 ,, | + | 0 |
| | 2 | 209 ,, | + | Papilloma of gut. |
| | 5 | 209 ,, | + | 0 |
| | 10 | 181 ,, | 0 | Cancer of breast. |
| | 8 | 216 ,, | + | Tumour duct. acust. ext. |
| | 6 | 218 ,, | 0 | 0 |
| | 7 | 267 ,, | 0 | Tumour duct. acust. ext. |
| | 9 | 274 ,, | + | Two cancers of breast, carcinoma of lung, tumour duct. acust. ext. |
| F. Basic | 4 | 232 ,, | + | Cancer of intestine. |
| | 1 | 244 ,, | + | Tumour of duct. acust. ext. |
| | 2 | 255 ,, | + | ,, ,, ,, ,, |
| | 5 | 279 ,, | + | ,, ,, ,, ,, |
| | 3 | 281 ,, | + | 0 |
| | 6 | 176 ,, | 0 | Cancer of breast. |
| | 8 | 183 ,, | 0 | Cancer of breast, tumour duct. acust. ext. |
| | 7 | 209 ,, | 0 | Ditto. |
| | 10 | 216 ,, | 0 | Retrobulbar carcinoma. |
| | 9 | 245 ,, | + | Cancer of breast, tumour duct. acust. ext. |

0 = no cancer; + = hepatoma. Benign cystic cholangiomata, present in nearly all animals, are not listed. Nos. 1–5 males, 6–10 females.

For controls to experiment C the reader is referred to tables of a previous paper (Bielschowsky, 1946), in which the results obtained in our strain of piebald rats after feeding the carcinogen for 25 weeks have been recorded. All Wistar rats of experiments D, E and F developed tumours before the end of the 40th week, with the exception of rat 6 (Exp. E), which died of an infection on the 218th day. As previously reported, 33 of 38 (25 females, 13 males) piebald rats which received the basic bread-milk diet developed cancer between the 21st and 36th week. Of the 5 remaining rats, which were all females, one died on the 281st day still free from tumours. In the other 4 rats cancers were found when they were killed on the 293rd day of the experiment.

The rate of survival was very much better in the yeast experiments. Two rats dying at an early date (Rats 3 and 6, experiment B) had to be omitted from the table. Of the 28 rats left, 22 were still alive at the end of the 42nd week; they seemed to be in excellent health and no tumours could be palpated; 14 of these were females and 8 males. However, neoplasms were found in 11 at the post-mortem examination. The commonest tumours were small hepatomas, having a diameter of 3–6 mm.; in one Wistar rat a small flat intraductal papilloma of the breast was discovered.

Histological examination of these early neoplasms frequently failed to give proof of their malignancy. A fairly large experience with the tumours induced by acetyl-amino-fluorene has taught, however, that all these early neoplasms were potentially malignant, and that they would have progressed in time to true cancers. 11 rats of the yeast series are listed in Table I as free from tumours. There was, however, one sign of the blastogenic action of acetyl-amino-fluorene—a few minute cystic cholangiomata. This type of benign tumour which we have never seen to progress to malignancy has been omitted from the tables, as in the earlier communications.

Table III records the body weights of the rats at the beginning of the experiments and on the day the carcinogen was withdrawn. There were no significant differences in gain of weight in any of the six experiments. There was, however, a difference in the liver weights between the animals receiving the basic and those

TABLE III.—*Average Weights of Rats (Groups of Five Animals).*

| | 4–7 weeks old. | First day of experiment. | 175th day. |
|---|---|---|---|
| Experiment A | Males | 72·8 g. | 168·4 g. |
| | Females | 52·6 g. | 151·2 g. |
| Experiment B | Males | 47·8 g. | 158·0 g.* |
| | Females | 44·6 g. | 129·5 g.* |
| Experiment C | Males | 53·6 g. | 195·0 g. |
| | Females | 57·8 g. | 139·0 g. |
| Experiment D | Males | 38·6 g. | 182·2 g. |
| | Females | 33·4 g. | 143·0 g. |
| Experiment E | Males | 56·6 g. | 190·2 g. |
| | Females | 70·0 g. | 148·0 g. |
| Experiment F | Males | 80·4 g. | 185·2 g. |
| | Females | 54·4 g. | 139·8 g. |

* Average of 4 animals.

fed the yeast diet. This difference was best seen when the average weights of the females of the piebald strain were compared. Only such animals were selected as were found to be free of hepatomas. The average weight of the livers of the females of experiment C was 7·9 g., of the controls 11 g. Previously Wilson, DeEd and Cox (1941) had observed a diffuse hyperplasia of this organ after feeding acetyl-amino-fluorene. Harris (1947) found this hyperplasia in 30 per cent of his animals. It seemed of interest that yeast prevented the hypertrophy of the liver as well as the development of hepatomas.

### DISCUSSION.

In all experiments reported in this paper the amount of carcinogen given was the same, the caloric intake similar and the percentage of bread varied only slightly. Any effects observed in the yeast series have, therefore, primarily to be attributed to the substitution of milk powder by dried yeast. Experiment E is in good agreement with Harris's findings that an increase in milk proteins failed to protect against the action of 2-acetyl-amino-fluorene. There exists now good evidence that a high casein-high riboflavin diet protects only against dimethyl-amino-azo-benzene. Even related compounds (Giese, Clayton, Miller and Baumann, 1946) are not inhibited to the same extent by such a diet. Further, Strong and Figge (1946), who reviewed the literature on this subject, did not succeed in inhibiting by a diet of liver supplemented by a combination of raw milk, riboflavin and xanthine the development of tumours induced by subcutaneous injection of methylcholanthrene. Harris's failure to protect against acetyl-amino-fluorene by a diet which inhibited the action of " butter yellow " has already been mentioned.

The results obtained in the yeast experiment are not impressive, but they seem significant since the same trend of delayed tumour growth was found in three experiments involving various litters of two different strains of rats. It would probably have been an advantage to use a smaller dose of the carcinogen in the experiments reported in this paper. A daily dose of 2 mg. of acetyl-amino-fluorene will still produce a high percentage of cancers.

It seems possible that the relatively low incidence of cancers observed by Cantarow, Paschkis, Stasney and Rothenberg (1946) after prolonged treatment of rats with acetyl-amino-fluorene was partly due to a supplement of brewers' yeast in the diet given.

The problem whether certain dietary factors are true anti-carcinogens, or protect only the liver against a damage the reaction to which leads to the establishment of cancers, could not be answered by workers who studied the effect of diet in rats receiving dimethyl-amino-azo-benzene. It was hoped that working with a more versatile carcinogen would give a clue to this question. At first sight it seems that in experiments A, B and C the development of all types of tumours was delayed. There was, for instance, a significant reduction in the incidence of mammary cancers in females of the Wistar strain. This finding, however, could be explained also by the assumption that a less damaged liver was better able to cope with an excess of endogenous oestrogen and so prevented the development of tumours of the breast. No tumours of the small intestine were found in experiment C—another fact which seemed to suggest a general anti-carcinogenic effect of the yeast diet. In unpublished experiments it has been found that the incidence of this type of tumour could be reduced by various unrelated agents. Therefore the adeno-carcinomata of the small intestine were not considered suitable material for deciding this question. The third type of tumour significantly affected was the squamous keratinizing carcinoma of the head, which is specially frequent in the piebald strain. Only three such tumours were found, one of very small size in a Wistar and two larger in piebald males, This last result could best be used as an argument in favour of a general anti-carcinogenic action of yeast. It is felt, however, that it is too early to draw such a conclusion. As long as it is not known whether or not acetyl-amino-fluorene is converted in the liver into an " active metabolite," an important fact necessary for a proper interpretation is missing, and any discussion of the role of dietary factors in the pathogenesis of the amino-fluorene induced tumours is better postponed.

### SUMMARY.

A diet containing 15 per cent dried yeast delayed significantly the development of cancers induced by 2-acetyl-amino-fluorene. The only neoplastic lesion which could not be prevented were minute benign cystic cholangiomata, which were found in all animals.

### REFERENCES.

BIELSCHOWSKY, F.—(1944) *Brit. J. exp. Path.*, **25**, 1.—(1946) *Ibid.*, **27**, 135.
CANTAROW, A., PASCHKIS, K. E., STASNEY, J., AND ROTHENBERG, M. S.—(1946) *Cancer Res.*, **6**, 610.
GIESE, J. E., CLAYTON, C. C., MILLER, E. C., AND BAUMANN, C. A.—(1946) *Ibid.*, **6**, 679.
HARRIS, P. N.—(1946) *Ibid.*, **6**, 487.—(1947) *Ibid.*, **7**, 88.
STRONG, L. C., AND FIGGE, F. H. J.—(1946) *Ibid.*, **6**, 466.
WILSON, R. H., DE EDS, F., AND COX, A. J.—(1941) *Ibid.*, **1**, 595.
DU VIGNEAUD, V., SPANGLER, J. M., BURK, D., KENSLER, C. J., SUGIURA, K., AND RHOADS, C. P.—(1942) *Science*, **95**, 174.